# Screening and identification of potential inhibitor for visceral leishmaniasis (VL) through computational analysis

**DOI:** 10.1186/s43141-022-00318-3

**Published:** 2022-02-23

**Authors:** N. Shaslinah, P. Sangavi, R. Sangeetha, S. Gowthamkumar, V. Sindhu, K. Langeswaran

**Affiliations:** 1 Cancer Informatics Laboratory, Department of Bioinformatics, Alagappa University, Karaikudi, Tamil Nadu India; 2 Department of Physics, Mannar Thirumalai Naicker College, Pasumalai, Madurai, Tamil Nadu India; 3 Faculty of Allied Health Sciences, Chettinad Hospital & Research Institute, Chettiand Academy of Research and Education, Kelambakkam, Tamil Nadu India

**Keywords:** Trypanothione synthetase, CASTp, Visceral leishmaniasis, Molecular docking, Molecular dynamics, Binding free energy, ADME prediction

## Abstract

**Aim:**

The aim of this investigation is to detect potential inhibitor for visceral leishmaniasis through computational analysis.

**Background:**

Leishmaniasis is categorized as a vector born pathogenic infection prevalent in tropical, subtropical, and in Mediterranean zones spread by intra-macrophage protozoa. The clinical syndrome of leishmaniasis is divided into the following type’s namely cutaneous leishmaniasis, mucocutaneous leishmaniasis, visceral leishmaniasis, and dermal leishmaniasis. Trypanothione synthetase is a key enzyme involving in glutathione biosynthesis as well as hydrolysis. Trypanothione is one of the promising drug targets for parasites. Parasites are inimitable with concern to their dependence on trypanothione to regulate intracellular thiol-redox balance in fighting against oxidative stress and biochemical anxiety. However, trypanothione synthetase was presumed as the target therapeutic alternate in VL therapy.

**Objective:**

The important objective of this current investigation is to identify or analyze the potential inhibitor for V. leishmaniasis through computational approaches which include virtual screening, molecular docking, ADME prediction, and molecular dynamic simulation.

**Methods:**

An investigation was performed to develop a 3D protein structure, using computational screening among associated similar structured proteins from popular compound database banks such as Specs, Maybridge, and Enamine, to detect novel staging with a series of validation for emerging innovative drugs molecules. Modeled protein ligand complex was further analyzed to know the binding ability of the complex. Molecular dynamics were performed to ascertain its stability at 50 ns.

**Results:**

Trypanothione synthetase overall ability in the outcome of series of analysis. Among three database compounds screened, the compound from the Specs database exhibited the better protein-ligand docking scores and fulfilled the drug-like properties through ADMET analysis, and the docked complexes had better stability throughout the simulation. Besides, the other two database leads fulfilled the pharmacological properties, and the complexes were stable in the simulation.

**Conclusion:**

By analyzing the various compounds from different databases, we concluded that the Specs database compound exhibits potential activity against the target protein and is considered a promising inhibitor for trypanothione synthetase.

## Introduction

Visceral leishmaniasis (VL) is a dangerous destructive syndrome, among different kinds of leishmaniasis. Other names of VL are Kala-Azar, black fever, or Dumdum fever [1]. VL is caused by the *Leishmania donovani* which is considered a fatal systemic disease if remained untreated. The characteristic features of VL transmission are two dissimilar ways. Zoonotic VL transmission is transferred through animals towards vector towards human and anthroponotic transmission is from human to vector to human. Unique clinical features of VL includes lengthen fever, a sudden weight loss, liver inflammation, and increased signs of anemia, resulting in more complications due to the following cosmopolitan infections. It intruded into the microphages and multiplied in it, to attack and target reticuloendothelial systems covering the liver, spleen, and bone marrows [2]. Albeit affirmed instances of VL have been accounted for from 66 nations, 90% of the world’s VL trouble happens on the Indian subcontinent and in Sudan. The existing pattern of leishmania is completed in two hosts: one is the vertebrate host, and another is the invertebrate host [3]. Ultimately, the mortality was caused by the fragile immunological capability of the infected patient. The metabolism of thiol-dependent redox, distinctive features of metabolism that differentiate trypanosomatids from human beings, provides constant biological drug target for the designing of potential medications. Trypanosomatids, flavoenzyme trypanothione reductase, and trypanothione are supported to the homeostasis of intracellular redox [4]. Trypanothione (bis(glutathionyl)spermidine, TS2—trypanothione’s oxidized form, T(SH)2—the compact type of trypanothione, could be a necessary molecule for modulating the parasite’s oxidative stress. Synthesis of trypanothione is catalyzed by dual significant enzymes: trypanothione synthetase (TryS) and trypanothione reductase (TryR). Two ATP molecules are provided energy which is employed to the spermidine from one molecule and glutathione from two molecules. TryS is accountable for the production of trypanothione. TryR maintains its reduced form T (SH) 2 in trypanothione using the NADPH as a cofactor. NADPH is often provided by the oxidative phase of the pentose phosphate pathway through glucose 6-phosphate dehydrogenase [5]. Trypanothione T (SH) 2 reduced forms are employed by the tryparedoxin/tryparedoxin peroxidase system (TXN/TXNPx) to reduce hydrogen peroxide, alkyl-hydroperoxide, and other reactive oxygen species produced by the macrophage [6]. TryR, TryS, and TXN/TXNPx are mandatory for the survival of parasites by protecting them against oxidative stress. Trypanothione pathway is absent in humans but necessary for the survival of parasites; hence, they are categorized as antileishmanial drug target [7]. Under these conditions, TryS remains the most promising target because leishmania is a low-abundance, essential enzyme because without any homologs of humans. The significance of TryS activity for the feasibility of the parasites has been proven in vitro and in vivo by genetic and pharmacological methods on *T. brucei* and *L. infantum*. A kinetic model of trypanothione T (SH)2 metabolism in *T. cruzi* predicted that to decrease the T(SH)2 synthesis by 50%, it is fundamental either to inhibit TryS by 63% or over 98% of TryR. TryS moderates inhibition; it seems that might be a promising target in drug development. Besides, TryS has several benefits as a molecular target candidate in drug development; it is encoded by a single copy gene; the TryS structure of L. major has been clarified, TryS has been publicized to afford metabolic regulator to the trypanothione pathway in *T. cruzi*, and kinetic evidence is obtainable for multiple TryS.

Spermidine and 2 glutathione is joined to form T(SH)2 (disulfide-hydroperoxidase). This formation catalyzes the enzyme name trypanothione synthetase. Next, it forms the T(SH)2 which catalyzes trypanothione reductase (TryR). The TS2 is converted into T(SH)2, and T(SH)2 is converted into TS2. So, it is called a reverse reaction. Again, T(SH)2 is reduced into tryparedoxin. A thiol redox reaction is involved that time; the mammal host is involved in different mechanisms, but the parasites do not have that mechanism; so, it is used as an inhibitor. Again, TS2 is converted into T (SH)2; this formation catalyzes the enzyme which is reductase; then, it converted into tryparedoxin which catalyzes the enzyme which is peroxidase; it leads to the antioxidant defense. In mammals, GSSG is converted into GSH. Both reactions are only inhibited by trypanothione synthetase. In the present investigation, it was aimed to find trypanothione synthetase inhibitors using virtual screening and molecular dynamic simulation techniques to evaluate their ability to be used as inhibitors to the specific VL disease. This current examination additionally engaged with homology demonstrating in Swiss port model and ADME property to locate the physicochemical property for the recognized potential compounds.

## Materials and methods

In this study, computational analysis had done on the Intel core TM 2, 160 GB hard disk, and Linux enterprise version 5.0 as OS. In this study, Uniprot, protein BLAST, structure analysis, and verification server; sitemap, GROMACS, virtual screening workflow, and glide module; and ADME prediction was carried out to find the potential inhibitor for leishmaniasis.

### Sequence analysis

Trypanothione syntheses were compared in the blast search using the BLASTp algorithm, which is used for aligning the target sequence against the PDB [8]. The BLAST result shows high identity and query coverage with the protein sequence database in the PDB database. Thus, we performed protein homology modeling in prime (Schrodinger suite) using trypanothione synthetase.

### Assessment of the model

For assessing the overall stereochemical quality of the modeled protein, SAVES [9] was used for structure analysis. PROCHECK program was used for stereochemical excellence of the modeled protein structure and overall structural geometry, and the structure was refined by the GALAXY [10] server. The simulated 3D model was evaluated for its stereochemical quality by Ramachandran plot using PROCHECK, and root-mean-square deviation (RMSD) value was noted. Prosa was utilized to obtain a *z*-score of Ramachandran plot; the verify 3D was used to determine the compatibility of an atomic model (3D) with its location and environment and comparing the result to the good structure.

### Molecular dynamics of trypanothione synthetase putative

Molecular dynamic (MD) simulations were done to analyze the stability of the modeled protein. MD is the computational simulation of the physical movement of atoms and molecules. GROMACS (GROningen Machine for Chemical Simulation) is a molecular dynamics package, which is assigned for simulations of protein, lipids, and nucleic acid. MD simulations were carried out using the GROMACS 4.6.3 package, GROMOS96 43a1 force field. Trypanothione synthetase **s**olvation was done in a cubic box and the SPC12 water model. Energy minimized complexes were subjected to 50 ns position restraining simulation to relieve close contact which included NVT and NPT equilibration phases also. During these equilibration phases, leap-frog integrator was used for 50 ns simulations. Coordinates, velocities, and energies were updated every 0.2 Ps with a LINCS algorithm to constrain bond lengths. Finally, 50 ns production phase MD was performed at the NPT canonical ensemble.

### Protein preparation

The planning wizard technique was used to design the protein structure. It involved a preparatory process and its refinements. During this event, parent carbon particles were put in with hydrogen (H2) molecules, whereas unnecessary water molecules were avoided and removed. Impact refinement module OPLS-2005 power field was applied to minimize, and it closed when RMSD reached out of 0.30 Å, due to it ensuring quality, vitality, and dependability for using further analysis [11].

### Virtual screening

This is a computational-based screening approach that helps to frame or design novel drugs using screening of a large number of chemical compounds, which is obtained from different databases. It is also helpful to find the active site of target receptors. The finding of active sites in target protein is a prime task and initiative point of virtual screening. Development of grid at the active site pocket was determined by the site map module [12]. Screening of chemical compounds was initiated using XP (extra precision) docking by employing a slide module. A computational search was performed to find possible conformations. Conjugate gradient (CG) minimization with steepest descent minimization along with a default value was found to be about 0.05 A at the initial, followed by 1.0 Å for reaching maximum extent. Based on the convergence grouping, energy charge and gradient criteria altogether were determined, and the default value was recorded to be 107 and 0.001 kcal/mol respectively. The abovementioned criteria were accounted for proceeding docking techniques, and glide score was considered to prioritize the best conformations to the selected ligands.

### Preparation of ligand

Ligands were filtered from three different databases (Enamine, Maybridge, Specs). Based on the highest glide score, glide energy, and some essential criteria, some of the ligands were selected, and those ligands were prepared using LigPrep modules using Merck molecular force field [13]. Ligand preparation using MMFF involved the development or conversion of 2D structure to 3D of ligand molecules in the optimized potential for liquid simulation for a field. By admitting hydrogen atoms, ligand bond orders were measured to neutralize. Minimization was also affected.

### Protein active site prediction

#### CASTp

To carry out design, identifying location spot and describing and assessing concave surface regions of the 3D structure of the selected proteins, the searching pockets were detected, and it was conceded or hidden in the inner side of the proteins. Surface accessibility of the pockets and unapproachable cavities were detected and determined using the CAST p server [14].

#### Prediction of MM-GBSA energy/binding energy

To compute the prime binding energy of the searched chemical compounds, it was performed by employing MM-GBSA. This method was carried out by adapting the OPLS-2005 force field along with the GBSA solvent model sourced in Schrodinger. To analyze solvent accessible surface area, we used surface generalized born model with Gaussian surface, alternate to the Vander walls surface which was admitted [15].

The binding energy (Δ G bind) was derived from

1 $$\Delta\ \mathrm{G}\ \mathrm{Bind}=\Delta \mathrm{E}+\Delta \mathrm{G}\ \mathrm{solv}+\Delta \mathrm{G}\mathrm{SA}$$

2 $$\Delta \mathrm{E}=E\ \mathrm{complex}-\mathrm{E}\ \mathrm{protein}-\mathrm{E}\ \mathrm{ligand}$$

Here, *E* denotes minimized energy value for protein-ligand complex, similarly

3 $$\Delta\ \mathrm{G}\ \mathrm{solv}=\Delta \mathrm{G}\ \mathrm{solv}\ \left(\mathrm{complex}\right)-\mathrm{G}\ \mathrm{solv}\ \left(\mathrm{ligand}\right)$$

where G solv indicates salvation free energy of the complex protein inhibitor.

4 $$\Delta \mathrm{GSA}=\mathrm{GSA}\ \left(\mathrm{complex}\right)-\mathrm{GSA}\ \left(\mathrm{protein}\right)-\mathrm{GSA}\ \left(\mathrm{ligand}\right)$$

where Δ GSA (complex) and Δ GSA (ligand) indicated surface area energies for the complex.

#### MD simulation

Selected target protein and lead compounds were subjected to examine molecular dynamic simulation measure, using GROMACs at 50 ns. The minimization of energy to the test complex was determined by the deepest descent algorithm and neutralized using position restrained dynamic simulation. At 50 ns, dynamic simulation of the complex was tried. The determinant characteristics such as hydrogen bond numerical, root-mean-square deviation (RMSD), and fluctuation (RMSF) were also measured by employing GROMACS [16].

#### ADME prediction

ADME prediction is the panel for finding the physicochemical and pharmacokinetic properties of the chemical compounds. The Qikprop module is used for finding the ADME properties of the lead selected compounds [17].

## Result

### BLASTp

Sequence similarity search for the given query sequence (Uniprot id: A0A3Q8IGF4) was done by using BLASTp (retrieve the protein sequence of the target from Uniprot in FASTA format and select the sequence (PDB ID_2VOB)) to predict the 3D structure of the target protein using the Swiss model server.

### Swiss model

**Fig. 1:**
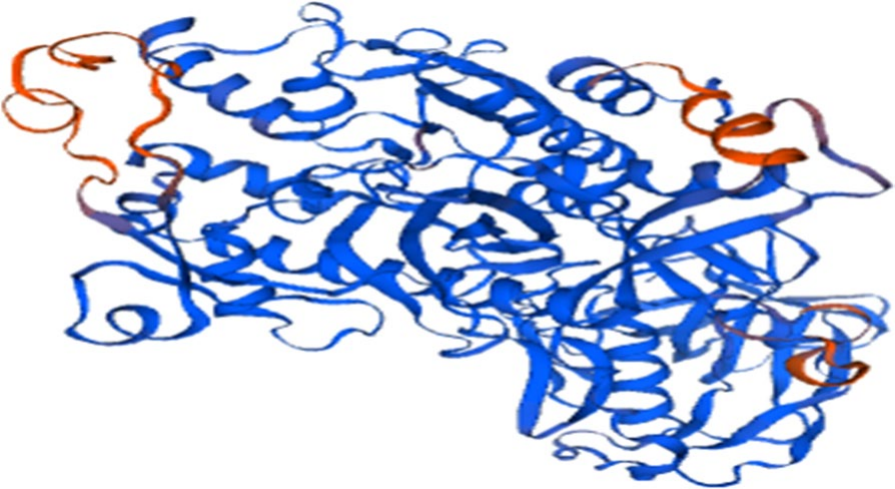
3D structure of modeled protein trypanothione putative synthetase, protein

It is a manifesto for contributing homology modeling to the unidentified protein structures. It is a module, providing a varied application in 3D reliable protein structure. This offers and helps to design protein structures to formulate target proteins associated with evolutionary lines (Fig. 1).

### Homology modeling and validation

**Fig. 2:**
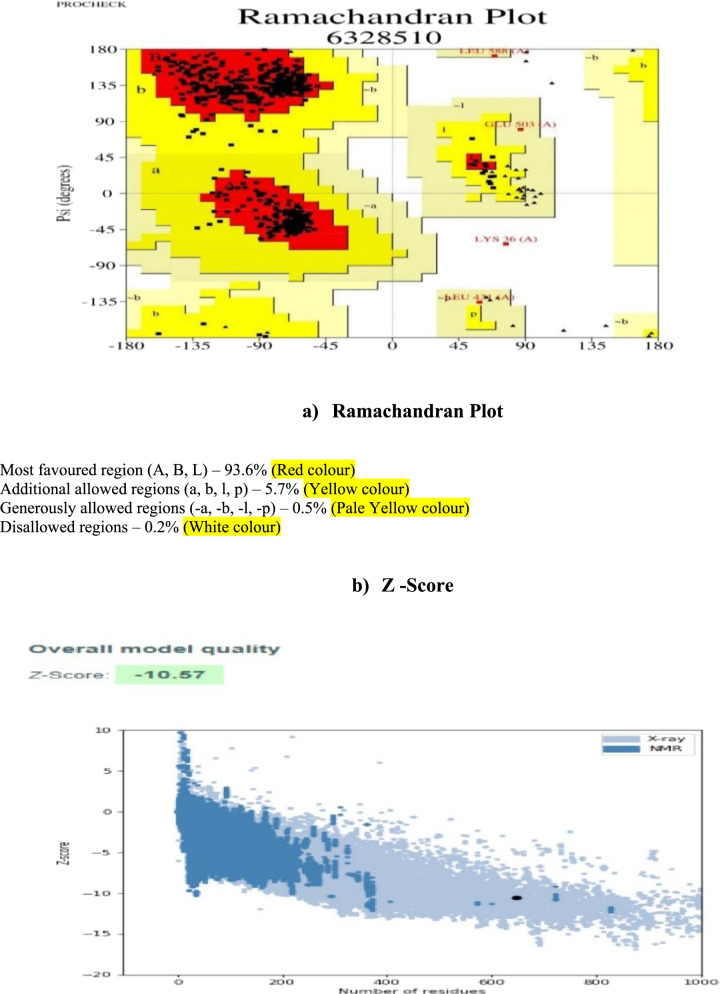
**a** Ramachandran plot analysis. **b** Z score analysis of modeled trypanothione synthetase

It is an effective theoretical model for determining the 3D structure for unspecified proteins. It can detect structure physical principal based on its energy and interaction. The invented model was scored using SAVE findings. Moreover, the Ramachandran plot illustrated and deciphered phi and psi angles for residual amino acids in the specified region of trypanothione synthetase (Fig. 2a, b). Our present work reveals that the plot showed 93.6% amino acid residue in favor spot, 5.7% was found in the allowed segment, and 0.5% was observed in generally allowed part, while 0.2% residues observed in the disallowed region.

### Analysis of the stability of the modeled trypanothione synthetase protein

**Fig. 3:**
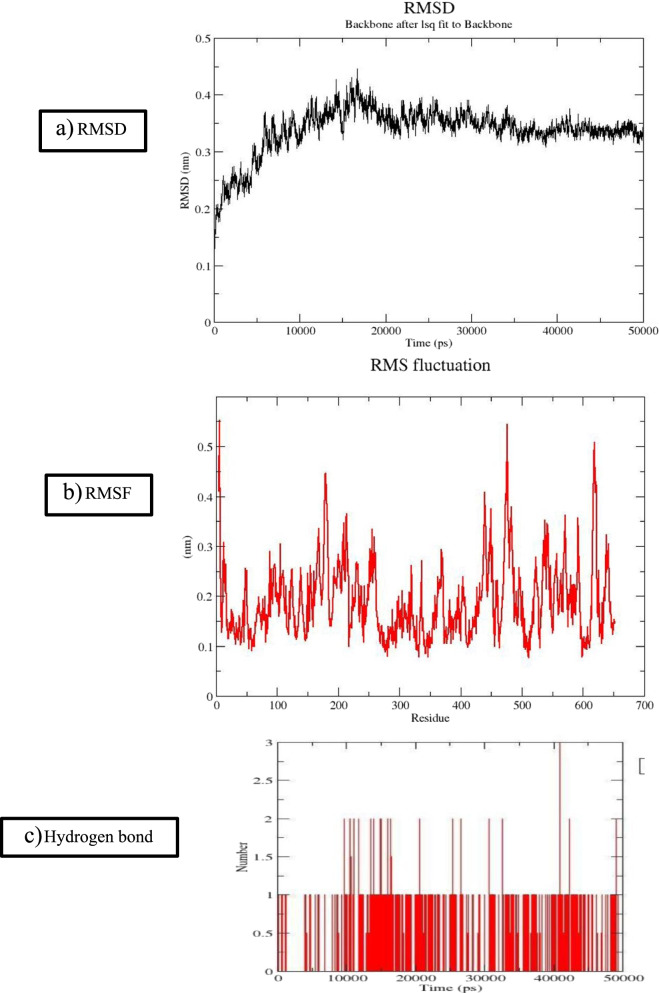
**a** RMSD, **b** RMSF and **c** hydrogen bond analysis of modeled protein—trypanothione synthetase

Trypanothione synthetase was targeted to evaluate dynamics nature at 50 ns. The potential energy has been presumed that it was stable in the MD process. The trajectory of trypanothione synthesize is known to be an involved assessment of RMSD and RMSF and for the determination of the stability of the protein (Fig. 3a–c). In the present study, the RMSD profile envisaged an average deviating was observed and reached powerful stability in the course of the simulation. Similarly, RMSF plot peaks exhibited modulation in the backbone; active cavity residue is very limited and was recorded. An increasing trend was observed in the RMSF of residues such as 450_600, due to the hidden occurrence at the loop structure. The study has been inferred that the presumed model protein of trypanothione synthesize was found to be a more powerful and stable compound. However, an average structure was taking the process further investigations.

### Protein active site prediction

#### CASTp

**Fig. 4:**
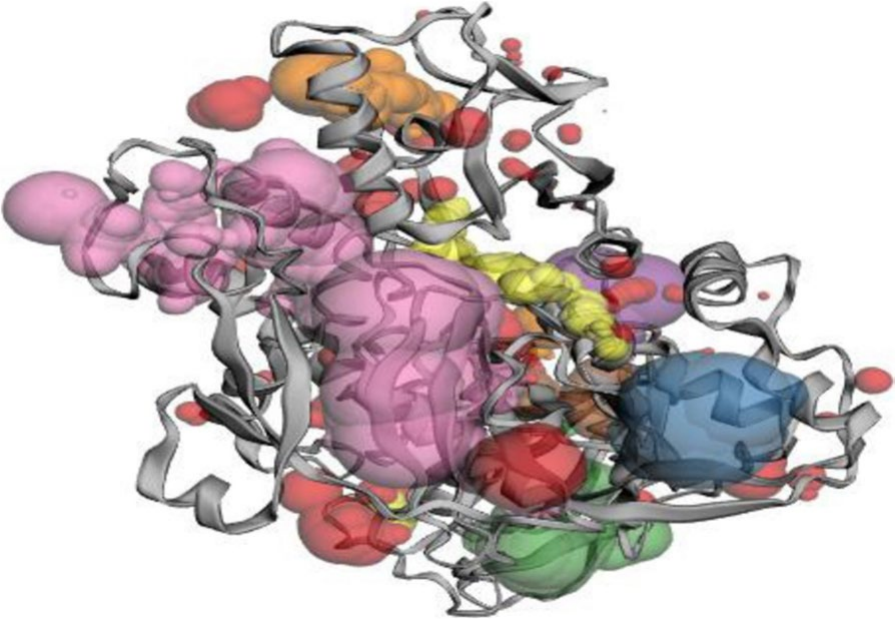
Active site of modeled protein obtained from the CASTp server

The active site (binding pockets) of modeled protein was predicted by using the online prediction tool CASTp. About 23 binding pockets were predicted for the protein; among them, the top four pockets were shown in Fig. 4 and Table 1. The binding cavity of the volume and area with 1392.832 and 1342.869, respectively, can be considered for further analysis. The amino acids residing in this cavity may play a major role in the binding of ligand molecules during docking analysis. In PDBsum, we elucidated the structure of the modeled protein; the protein have 10 sheets; 2 beta alpha bête units, 14 beta hairpins, 13 beta bulges, 35 strands, 20 helices, 22 helix-helix interactions, 65 beta turns, and 5 gamma turns were identified.

**Table 1:** Site score and volume of the site obtained through CASTp server

Pocket ID	Area	Volume	Negative volume color	Representation style
1	1392.832	1342.869	Pink	Cartoon
2	347.208	512.034	Blue	Cartoon
3	608.890	451.738	Green	Cartoon
4	116.118	165.531	Purple	Cartoon
5	246.083	125.848	Orange	Cartoon
6	196.744	51.240	Yellow	Cartoon
7	140.018	37.732	Brown	Cartoon
8	87.523	37.463	White	Cartoon
9	54.450	34.640	Red	Cartoon
10	34.640	31.260	Red	Cartoon

#### Virtual screening

**Fig. 5:**
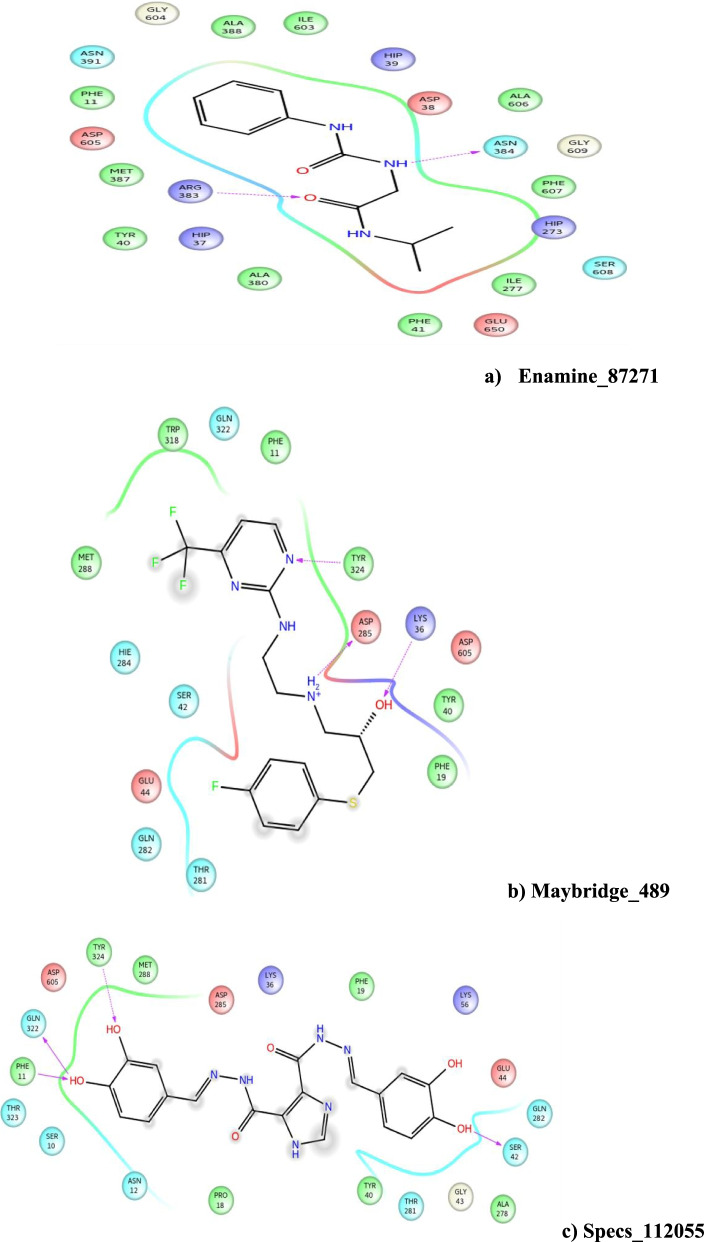
Docking analysis of lead compound interaction with trypanothione synthetase

The hierarchical complex screening procedure was performed to identify novel inhibitors against trypanothione synthetase protein. Enamine, Maybridge, and Specs database compounds were screened against trypanothione synthetase modeled protein [18]. The screened compounds were filtered out with LigPrep modules. The residues present in the active site are TYP_324, ASP_285, LYS_36, ARG-383, ASN-384, TYR-324, GLN-322, PHE-11, and SER-42. Grid box was constructed at the dimensions of *X* = − 4.51, *Y* = − 26.5, and *X* = − 9.08. These residues were selected for the screening analysis; the active site was generated based on the co-crystallized ligand present in the trypanothione synthetase protein structure. The lead compound from the Specs database showed the highest docking score of about − 8.702 kcal/Mol, the Maybridge database compound has the highest docking score of about − 6.000 kcal/Mol, and the Enamine database compound showed the highest docking score of about − 6.490 kcal/Mol (Fig. 5 and Table 2). The hydrogen bond interactions are also crucial for the protein-ligand binding and the stability of the complexes.

**Table 2:** Summary of the docking score of the top three compounds obtained from each database

Compound ID	Docking score (kcal/mol)	Interaction residues
Maybridge_489	− 6.000	TYP_324,ASP_285, LYS_36,
Enamine_87271	− 6.490	ARG-383, ASN-384
Specs_112055	− 8.702	TYR-324,GLN-322,PHE-11,SER-42

#### Binding free energy calculation

The protein-ligand complex was evaluated by using the related post-scoring approaches in MM-GBSA. The binding energy of the lead hits is listed in Table 3. The binding strength is determined by binding free energies of the compounds; Maybridge, Enamine, and Specs databases were − 55.951, − 52.317, and − 58.666 Kcal/mol respectively, which defines as the high binding affinity of the ligand towards the protein to inhibit the biological activity.

**Table 3:** Binding free energy calculation of the protein ligand complex using MM/GBSA methods

Compound ID	ΔG bind (Kcal/mol)	Interaction residues
Maybridge_489	− 55.951	TYP_324, ASP_285, LYS_36,
Enamine_87271	− 52.317	ARG-383, ASN-384
Specs_104059	− 58.666	TYR-324, GLN-322, PHE-11,SER-42

#### ADME prediction

The selected compounds were examined for absorption, distribution, metabolism, and excretion using the Qikprop module [19, 20]. The Qikprop module containing 44 relevant pharmaceutical descriptors which include hydrogen bond donor/acceptor, octanol/water partition coefficient, Rule of Five, human oral absorption, and molecular weight. The result suggested that the selected compounds have drug-like properties or pharmaceutical relevant properties and pharmacokinetic properties. The ADME properties of four compounds were tabulated (Table 4).

**Table 4:** The calculation of the ADME properties of compound

Compounds id	M/W	Donor HB	Acceptor HB	Qplog pw	Qplog po/w	Qplog HERG	Qpp caco	Qpp MDCK	Rule of 5	Rule of 3	Human oral absorption (%)
Maybridge_ 489	390.4	3.000	6.200	12.126	3.624	− 7.163	336.989	2061.0	0	0	93
Enamine_87271	235.2	2.250	3.750	13.319	1.173	− 2.791	309.798	406.122	0	0	78
Specs_104059	424.3	6.000	8.500	20.933	0.686	− 7.082	3.028	0.937	1	1	100

#### Molecular dynamic simulation

In the molecular dynamic simulation process to examine the stability of the protein-ligand complex, this level of water molecules was scrutinized. The simulation was performed usually according to the docking score and the interaction ability of the protein-ligand complex fixed at 50 ns. In our study, the RMSD plot technique exhibited that the selected lead compound Specs_112055 contains a good stability score and found between the ranges of 20–50 ns. RMSF plot results showed a pattern of peaks symptomatic to the rate of modulations of residues of protein and eluted with the best range of 550–600 residues. However, it would not interrupt the overall stability of the identified structural protein. A compatible hydrogen band affinity throughout the recreation time limit provides the quickest clue, on its tendency with regards to protein-ligand complex.

**Fig. 6:**
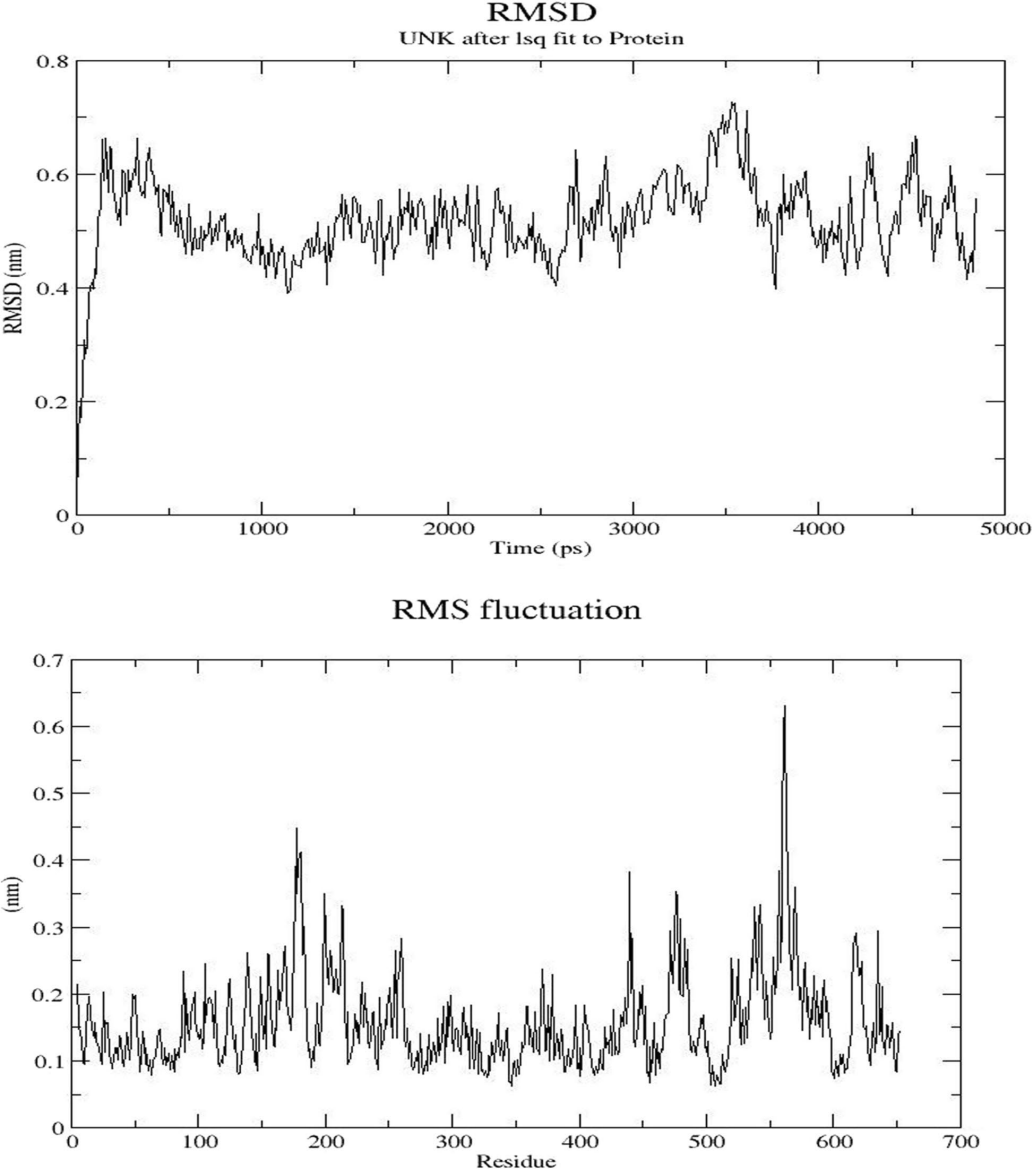
RMSD and RMSF analysis of lead compound (Specs_112055) and the target protein (trypanothione synthetase)

In a molecular dynamic study, water molecules were used to determining and checking the conformational stability of the trypanothione synthetase modeled protein. The potential energy has been presumed that it was stable in molecular dynamic simulations (MD) study in the range of 50 ns (Fig. 6a, b). The trypanothione synthetase was involved in the assessment of RMSD and RMSF, to confirm the stability of the protein. The RMSD plot shows that the trypanothione synthetase has good stability denoted during the range of 20 to 50 ns. During the period of the simulation, with the help of RMSF, the average oscillations of the macromolecular target protein at the residue level. The peaks of the RMSF plot indicate the final range of fluctuation in the modeled protein within the residues. The results conclude that the fluctuation rate of the trypanothione synthetase modeled protein gives 450–600 residues, with good stability due to the hidden occurrence at the loop structure. The study finally concludes that chosen trypanothione synthetase protein was found to be more stable and make a powerful complex for the further future drug discovery process. However, an average structure was taking the process further investigations.

## Discussion

Trypanothione synthetase (TryS) is one of the essential enzymes for leishmania’s survival, so it is recognized as the important drug target for drug designing [21]. TryS is the important enzyme that is normally involved in trypanothione synthesis. It also is responsible for maintaining the polyamines level; the polyamines are an essential factor for cell differentiation and proliferation. In this study, we target TryS, to design anti-leishmanial drugs with the help of different databases. We performed sequence analysis for modeling the unknown structure of the target protein. We filtered out the lead compounds, which were screened against the target protein. Structure-based virtual screening is one of the leading techniques to develop antileishmanial drugs [22]. The virtual screening which is based on molecular docking is one of the essential factors for finding the potential inhibitor for leishmaniasis, which helps to find out the protein-ligand interactions and protein-protein interactions which were monitored by an array of intermolecular interactions [23]. Besides, we performed docking analysis to find the best-docked complex. The pharmacological properties of the lead compounds also proved that they are eligible for a drug candidate, which is proven by ADME properties. The results showed that the lead compounds from various databases exhibit their potential, as a promising inhibitor for visceral leishmaniasis.

## Conclusion

The dataset compounds shown the interaction with Maybridge (TYP_324, ASP_285, and LYS_36), Enamine (ARG_383, ASN_384), and Specs (TYR-324, GLN-322, PHE-11, SER-42) amino acid residues, while the selected compounds interact with nitrogen, hydrogen, OH, oxygen, HO, and amino acid residues. From this result, it is tangible that the partitioned compounds are docked to target protein. The screened compounds specs_112055 have a high docking score, high binding energy, and strong interaction with the binding cavity of the target protein. In the analysis, Specs_112055 is the promising lead compound to inhibit the activity of visceral leishmaniasis targeted protein.

## Data Availability

Not applicable.

## Abbreviations

VL: Visceral leishmaniasis
TryS: Trypanothione synthetase
TryR: Trypanothione reductase
BLAST: Basic Local Alignment Searching Tool
OPLS: Optimized Potentials for Liquid Simulations
PDB: Protein Data Bank
XP: Extra precision
MM-GBSA: Molecular mechanics generalized born surface area
CASTp: Computed Atlas of Surface Topography of proteins
GROMACS: GROningen MAchine for Chemical Simulations
SAVES: Structure Analysis and Verification Server
ADME: Absorption, distribution, metabolism, excretion
RMSD: Root-mean-square deviation
RMSF: Root-mean-square fluctuation
MD: Molecular dynamics
